# Laryngeal candidiasis: Our experience from sixty biopsy specimens

**DOI:** 10.1111/coa.13034

**Published:** 2017-12-27

**Authors:** A. Yao, T.J. Bates, J. Pearson, M. Robinson, C. Ward, J. Powell

**Affiliations:** ^1^ Department of Otolaryngology, Head and Neck Surgery Stepping Hill Hospital Stockport UK; ^2^ Department of Cellular Pathology Royal Victoria Infirmary Newcastle Upon Tyne UK; ^3^ Institute for Cell and Molecular Biosciences Newcastle University Newcastle Upon Tyne UK; ^4^ Institute of Cellular Medicine Newcastle University Newcastle Upon Tyne UK; ^5^ Department of Otolaryngology, Head and Neck Surgery Freeman Hospital Newcastle Upon Tyne UK

**Keypoints**

- Candidiasis was found in a number of laryngeal biopsy specimens tested. These patients had no reported visual evidence of candidiasis on laryngoscopy.
- Laryngeal candidiasis was more commonly identified in the pathology samples of those with documented use of inhaled corticosteroid therapy (20%) than those without documented use (10%).
- Local discussion between otolaryngologists and pathologists on the testing of laryngeal samples for candidiasis is required to ensure appropriate investigations are performed in a standardised manner.

## 1 INTRODUCTION

Persistent throat symptoms, such as dysphonia, globus and throat pain, are highly prevalent and are a significant cause of morbidity.^1^ In a number of cases, a clear cause of these symptoms is not identified, and many patients are treated empirically with lifestyle advice and/or anti‐reflux medication.^2^

There is an increasing frequency of respiratory diseases, such as asthma and chronic obstructive pulmonary disease (COPD), with associated increased use of inhaled corticosteroids (ICS).^3^ Oropharyngeal candidiasis is a well‐recognised complication of ICS, particularly when inhaler technique and oropharyngeal hygiene are poor.^4^ There is, however, limited evidence on the prevalence of laryngeal candidiasis in those taking ICS.^5^ While clinical diagnosis is sometimes possible, it has been highlighted in oropharyngeal candidiasis that clinical findings do not always correlate with the presence of fungi.^6^, ^7^

We hypothesised that laryngeal candidiasis may be an under‐recognised cause of laryngeal inflammation and persistent throat symptoms in a subgroup of patients presenting to ENT clinics, particularly those taking ICS. We therefore aimed to retrospectively review the presence of candidiasis in a series of laryngeal biopsies.

## 2 METHODS

We retrospectively reviewed the laryngeal biopsy samples and case notes of patients presenting to the ENT outpatients department of the Newcastle upon Tyne Hospitals NHS Foundation Trust. We identified a series of patients who had previously undergone laryngeal biopsy for persistent throat symptoms and had suspicious findings on flexible endoscopy and had documented current ICS use. We also identified from the same database of biopsy samples an age and sex‐matched group of patients without respiratory co‐morbidities or documented ICS use.

Patients were included if (i) there was no visual evidence of candidiasis documented following laryngeal examination under general anaesthesia (EUA) and (ii) the biopsy samples demonstrated no evidence of epithelial dysplasia or malignancy.

For patients fulfilling the study inclusion criteria, the formalin‐fixed paraffin‐embedded tissue samples were retrieved from the pathology archive, and three 4 μm sections were cut into superfrost microscope slides. The sections were then stained with diastase periodic acid schiff (DPAS) reagent to identify fungal hyphae. The DPAS staining was carried out in a clinical pathology accreditation (CPA) endorsed cellular pathology laboratory. A specialist head and neck pathologist (TJB or MR) then reviewed the sections and reported on any evidence of candidiasis.

### 2.1 Statistical analysis

All clinical data were sorted on secure NHS computers, and analysis was performed on Microsoft Excel 14.6.5 (Redmond, Washington, USA).

### 2.2 Ethical considerations

The project was registered with the Clinical Governance Department of the Newcastle upon Tyne Hospitals NHS Foundation Trust. Jason Powell was funded by a clinical research fellowship: WT108768MA Wellcome Trust.

## 3 RESULTS

Thirty patients were identified who had documented use of ICS and met our inclusion criteria (mean age 60 ± 4.6 years old [95% confidence interval]; male:female ratio 7:23). We identified 30 age and sex‐matched patients without documented respiratory co‐morbidities or ICS use, who had biopsies taken between January 2010 and July 2015. The most common indication for biopsy for patients in both groups was for non‐specific signs of inflammation (ICS group 15 of 30, non‐ICS group 14 of 30). The rest were for other benign causes, such as cysts or Reinke's oedema. Of the patients with documented ICS use, 15 of 30 initially presented primarily with dysphonia, 5 of 30 with persistent sore throat and 4 of 30 with globus. The remaining 6 of 30 presented with a combination of other symptoms, such as cough. The respiratory co‐morbidities included 16 of 30 with COPD, 11 of 30 with asthma and the remaining 3 of 30 had a combination of the two or bronchiectasis.

In the ICS group, only 2 of 30 previously had a DPAS staining performed by the pathology laboratory. Both were found to be negative, as in our assessment. Review of the pathology request cards demonstrated that only one patient had a respiratory co‐morbidity documented and none had the use of ICS documented by the requesting clinician.

**Figure 1:**
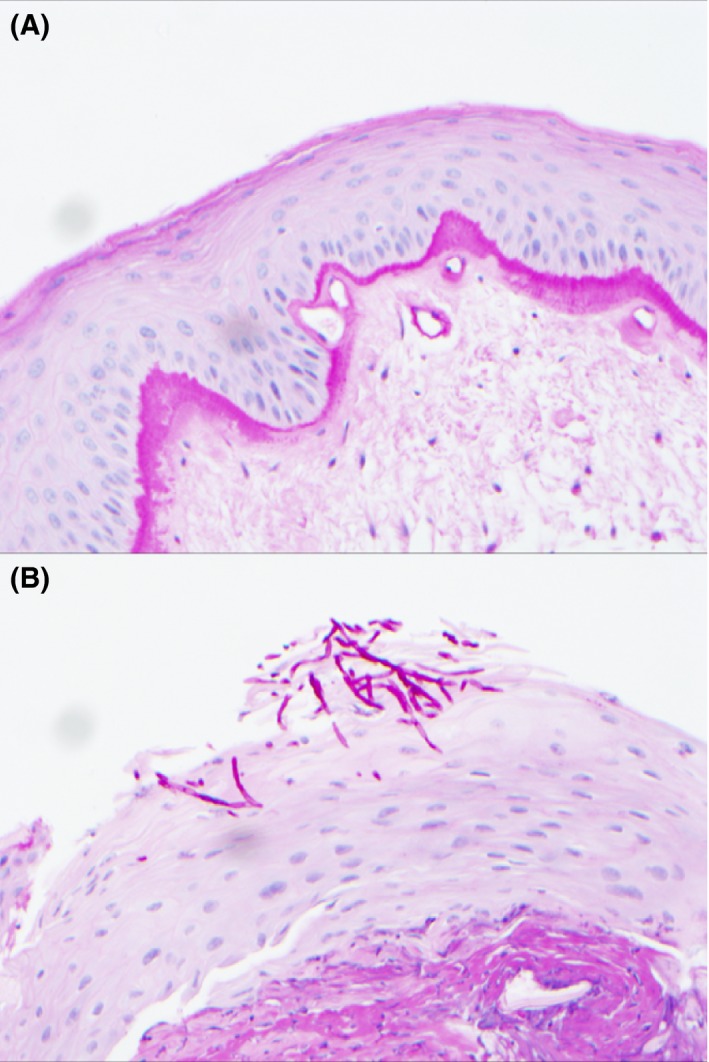
Laryngeal vocal cord biopsy stained with Diastase Periodic Acid Schiff (DPAS) at ×20 magnification. A, DPAS negative sample without fungal hyphae (×20 magnification). B, DPAS positive sample with magenta‐coloured fungal hyphae

Our DPAS testing of laryngeal samples demonstrated that overall 9 of 60 patients (15%) had evidence of fungal hyphae, consistent with candidiasis (see Figure 1). Candidal hyphae were found more frequently in those taking ICS (6 of 30, 20%), compared with the age and sex‐matched patients not taking ICS (3 of 30, 10%, 3 were all female).

## 4 DISCUSSION

### 4.1 Synopsis of key findings

Candidiasis was a surprisingly common finding in our series of laryngeal biopsy samples. These patients had no indication of laryngeal candidiasis on EUA and no evidence of malignancy. Candidiasis was apparently more common in patients taking ICS, when compared to age and sex‐matched controls. Laryngeal candidiasis may represent a potentially treatable cause for persistent throat symptoms in some patients. However, further studies would be required to fully assess whether the candidiasis caused these symptoms.

Notably, almost all of the biopsy samples taken from patients with ICS use were not initially tested for candidiasis by the pathology laboratory. Disclosure of ICS status or respiratory co‐morbidities was almost universally absent from the clinical documentation in the pathology request form.

### 4.2 Strengths and weaknesses of the study

This is a small retrospective study and subjects to several limitations. Firstly, the documentation of ICS may be unreliable, and it is conceivable that some of the non‐ICS use group were actually ICS users. DPAS is used for visualising the carbohydrate components of candida and as with any interpretive test will have limitations and the potential for reporter error. There are no guidelines on how many sections to stain when performing DPAS testing. DPAS is able to identify the glycoprotein content of fungi, although it may not be able to distinguish small early fungal stages due to their size or indeed specific candida species. The samples were all tested in a quality‐assured CAP‐endorsed pathology laboratory and reported by a specialist head and neck pathologist.

We present a relatively elderly cohort of patients reflecting the indications for laryngeal biopsies and need to exclude malignancy. There is a possibility post‐menopausal hormone changes may affect candida colonisation on laryngeal mucosa secondary to pH influences. This limits our conclusions with regard to the incidence of laryngeal candida in younger ICS users.

We were also unable to perform subgroup analysis of the EUA findings (such as inflammation or cysts) and the presence or the absence of laryngeal candidiasis due to the small study numbers. Similarly, we are not able to distinguish in this study whether throat symptoms are specifically due to candida infection or due to other benign pathologies such as Reinke's oedema or cysts. Furthermore, it would be ideal to compare the prevalence of candida in a control group of ICS users without throat symptoms. However, we would not routinely be taking laryngeal biopsies from normal larynx in patients without such symptoms. Observational evidence from this pilot study will assist in planning studies that further consider asymptomatic patients, adequate power and causality rather than correlation.

### 4.3 Comparison with other studies

The potential of laryngeal candidiasis to cause persistent throat symptoms is supported by Wong et al^8^ who reported that dysphonia was present in as many as 37 of 54 (69%) of patients with diagnosed laryngeal candidiasis. Turan et al^9^ reported a similar rate of laryngeal candidiasis to our study of 17.9% in 39 patients using ICS. From the oropharyngeal candidiasis literature, a study of 15 patients taking ICS for asthma found rates as high as 50% for pharyngeal yeast‐positive culture.^6^ Supporting our findings, it was noted that the presence of yeast did not always correlate with the clinical findings. Furthermore, in a review of 223 oral lesion biopsies, 4.7% of biopsies were positive for fungi on DPAS staining despite a lack of clinical signs of candida.^7^

### 4.4 Clinical applicability of the study

We have identified notable rates of laryngeal candidiasis, via DPAS testing of pathology samples, in a cohort of ICS users, and also high rates in those without a clear documentation of ICS use. These patients presented to ENT clinics with persistent throat symptoms and endoscopic findings of inflammation, without evidence of malignancy. It could be hypothesised that their symptoms may have been caused by this infection. However, there are many causes of persistent throat symptoms, not least ICS themselves, which are known to cause laryngeal irritation and inflammation.^5^ A larger, adequately powered, study assessing response to treatment is required to confirm the findings from our case series and investigate causality.

There are several key questions to answer in future studies. These include: does ICS cause laryngeal candidiasis, does laryngeal candidiasis causes throat symptoms, what is the effect of fungal stage on laryngeal inflammation and what is the effect of topical antifungal treatment on throat symptoms in ICS users? Furthermore, an important question remains regarding the impact of proton pump inhibitors on laryngeal candidiasis. Proton pump inhibitors appear to have a direct inhibitory effect of the H+‐ATPase enzyme activity native to fungi.^10^ They also have an indirect pH effect via alkalinisation, which affects the local aero‐digestive micro‐environment. These effects are likely to have impacts on both candida growth/colonisation and candida morphogenesis, by favouring a potentially pathogenic hyphal stage. Finally, it is yet not fully defined what the role of systemic antifungal treatment is in laryngeal candidiasis. Our experience with oesophageal candidiasis suggests that it does indeed have a role, especially in immunocompromised individuals. Given the increasing rates of ICS use worldwide and the socio‐economic impact of persistent throat symptoms, identification of a potentially treatable cause would be highly advantageous.^2^, ^3^

An important consideration is that routine histological practice in our unit is not to perform DPAS testing without clinical or histological indications of potential candidiasis. While the practice is variable amongst pathologists, it is crucial for otolaryngologists to document clinical suspicion of candidiasis and/or ICS use on pathology request forms to ensure full testing of laryngeal samples. Development of local and national guidelines for when to perform DPAS histochemistry would also be of benefit. Furthermore, we recommend spacer units for patients taking ICS to reduce the rate of candidiasis in the upper aero‐digestive tract. Greater understanding of the incidence of laryngeal candidiasis could aid clinical decision‐making which justifies further studies on this topic.

## CONFLICT OF INTEREST

None.
